# Polychromatic Virtual Retinal Imaging of Two Extended-Depth-of-Focus Intraocular Lenses

**DOI:** 10.1167/tvst.14.12.33

**Published:** 2025-12-31

**Authors:** Damian Mendroch, Stefan Altmeyer, Uwe Oberheide

**Affiliations:** 1 Institute for Applied Optics and Electronics, TH Köln University of Applied Sciences, Cologne, North Rhine-Westphalia, Germany

**Keywords:** intraocular lens, cataract, ray tracing, simulation, lens metrology, extended depth of focus (EDoF), mathematical modeling

## Abstract

**Purpose:**

This work characterizes two extended-depth-of-focus (EDoF) intraocular lenses, the Alcon IQ Vivity and the Bausch & Lomb LuxSmart, using virtual retinal imaging. A simulation-based examination tests these lenses under various conditions, while including dispersion and color effects.

**Methods:**

A custom sequential Monte-Carlo Ray Tracer simulates the propagation of a broadband daylight spectrum through a mathematical eye model. Realistic color images of a pinhole and resolution chart were generated, with variations in object distances and pupil size. A comparison is made with the monofocal Alcon IQ to highlight the benefits of EDoF models.

**Results:**

Simulated images clearly demonstrate the superior acuity and reduced aberrations of these lenses at intermediate vision. There are notable differences between both models: the LuxSmart lens exhibits an increased depth-of-focus under both mesopic and photopic conditions. Conversely, the IQ Vivity lens shows minimal aberrations for far vision under mesopic conditions, making it ideal for night-time driving, albeit with a lesser depth-of-focus compared to the LuxSmart.

**Conclusions:**

Our in silico investigation proves to be a valuable tool for evaluating intraocular lens performance. This study underscores the importance of characterizing lenses under both photopic and mesopic conditions and highlights the impact of chromatic aberration and color vision.

**Translational Relevance:**

The simulation-based approach expands the range of methods for assessing intraocular lenses. By providing realistic images, it allows both ophthalmologists and patients to more intuitively understand the aspects of these lenses. The simulation derives visual predictions from a purely mathematical model, thus bridging the gap from technology to clinical application.

## Introduction

Extended-depth-of-focus (EDoF) intraocular lenses (IOLs) offer a new, promising solution for cataract surgery. By enhancing intermediate vision, they serve as a middle ground between monofocal and multifocal lenses. Although their range of vision does not match that of multifocal intraocular lenses (MIOLs), they outperform MIOLs for intermediate vision under mesopic conditions.^1^^,^^2^ They also offer reduced photic phenomena compared with bifocal MIOLs^1^^,^^3^ and trifocal MIOLs,^3^^,^^4^ and improved contrast sensitivity compared to a trifocal IOL.^1^^,^^2^ This benefit is particularly evident with non-diffractive EDoF lenses, which show significantly fewer visual disturbances than their diffractive counterparts.^5^^–^^7^ EDoF IOLs are expected to have a higher tolerance to refractive shift and postoperative residual errors.^8^^–^^10^ Furthermore, some patients who are not suitable candidates for MIOL now have an opportunity to reduce their spectacle dependence.^11^^,^^12^ This is especially beneficial as intermediate vision plays an important role in daily activities.

The selection of an appropriate lens should take into account medical factors as well as the patient's preferences. This process necessitates a comprehensive understanding of the various lens types, as well as differences between specific models. A comparison of lenses, along with an estimation of photic phenomena and visual quality, would greatly aid in selecting a lens that meets the patient's needs. Typically, such decisions are based on a combination of clinical studies, publications with in vitro measurements, or, possibly biased, marketing material. In contrast to these three, this work introduces a simulation approach that facilitates virtual retinal imaging to demonstrate the lens performance across different scenarios. Some advantages of this approach include easier adjustment, higher reproducibility, more flexibility, and the elimination of the need for specialized equipment.

When simulating optical performance, a monochromatic characterization with a narrowband spectrum is insufficient. Real vision is strongly polychromatic, and the human eye exhibits significant levels of both longitudinal chromatic aberration (LCA) and transverse chromatic aberration (TCA). LCA is quantified as 2.5 diopter (D) over the entire visible spectrum,^13^^,^^14^ whereas the average magnitude of subjective foveal TCA is reported as 0.82 arcmin.^15^ Multiple works show decreased contrast sensitivity and visual acuity for broadband stimuli compared with narrowband sources.^16^^–^^18^ Roorda et al. directly demonstrated an improvement of acuity and contrast sensitivity with LCA and TCA being corrected for polychromatic light.^19^ Moreover, several studies show LCA increasing the depth of focus.^17^^,^^20^^,^^21^

In addition to aberrations, color contrast plays a significant role in the perception of disturbing effects and image sharpness. The complex interplay between color and luminance, influenced by psychophysical phenomena, such as the Hunt effect, the Bezold-Brücke shift, or the Helmholtz-Kohlrausch effect, further complicates visual appearance.^22^^,^^23^

It is noteworthy that there have been recent efforts to compare polychromatic IOL performance in vitro.^24^^–^^28^ Notably, the updated International Organization for Standardization (ISO) 11979-2:2024^29^ standard for in vitro measurements now also mandates a broadband white spectrum for optical characterization. In the following sections, an in silico approach is introduced that accounts for a wide spectrum and the chromatic behavior of all optical elements.

## Methods

### Lenses

This work compares three different lenses, one monofocal and two refractive EDoF IOLs. All three evaluated IOLs are biconvex, hydrophobic, and have a common refractive power of 20 D.

The monofocal AcrySof IQ (Alcon Laboratories Inc., Fort Worth, TX) with its aspheric design serves as a reference lens. The AcrySof IQ Vivity (Alcon Laboratories Inc.) incorporates the X-Wave-technology, a patented non-diffractive wavefront-shaping design^30^ that extends the depth of focus to 1.5 D.^31^ This effect is achieved through an elevated surface region between the radial position *r* = 0*.*55 mm and *r* = 1*.*1 mm on the anterior surface.^32^ Both Alcon lenses share a refractive index of *n* = 1*.*5542 and an Abbe number of *V* = 37.^31^^,^^33^ The second EDoF IOL, the LuxSmart (Bausch & Lomb Inc., Bridgewater Township, NJ), features an innermost zone on the anterior side that generates a combination of fourth- and sixth-order spherical aberration of opposite signs.^31^ This is followed by a transition region and an outer aberration-free zone. The EDoF of the LuxSmart is specified as 1.5 D^31^ and its optic material as *n* = 1*.*540 and *V* = 43.^31^^,^^34^

**Figure 1.**
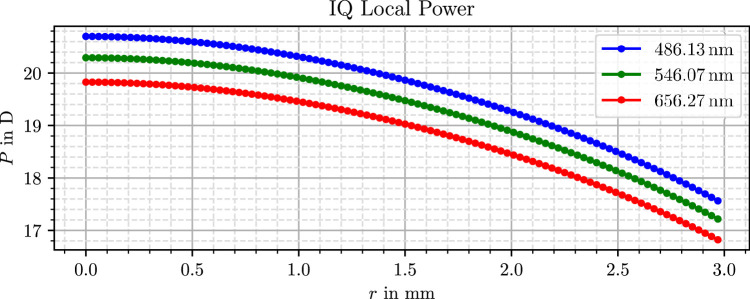
Simulated radial optical power profile of the Alcon IQ lens.

**Figure 2.**
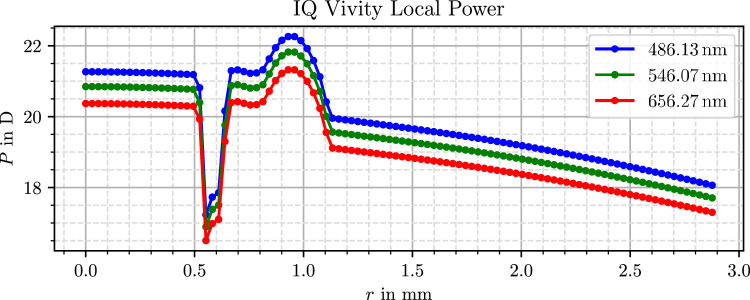
Simulated radial optical power profile of the Alcon IQ Vivity lens.

**Figure 3.**
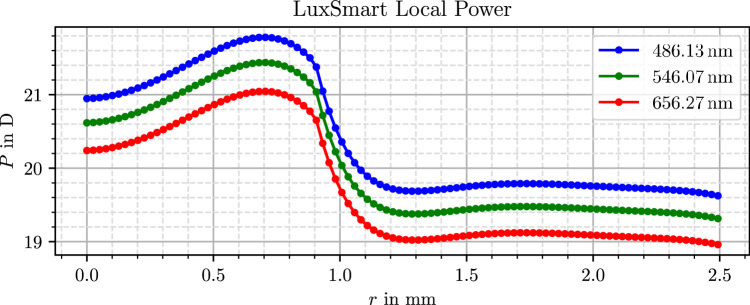
Simulated radial optical power profile of the Bausch & Lomb LuxSmart lens.

The virtual lens models used in this study originate from our previous work, where they were generated through a three-dimensional measurement and subsequent data processing. Further details on the measurement and processing procedure are available in Mendroch et al.,^35^ whereas a second publication^32^ presents the resulting lens properties, surface profiles, and refractive power distributions. For clarity and accessibility, the power profiles from these results are also presented in Figures 1, 2, and 3.

### Simulator

The custom-built simulator enhances the sequential raytracer from the previous work.^32^ Its features include the ability to model various spatial, spectral, and angular light distributions, as well as different types of dispersive and non-dispersive elements such as lenses, filters, apertures, and detectors. A new feature is the capability to render intensity and full-color images on a detector area. This is accomplished by propagating the entire visible wavelength spectrum toward the detector and rendering its spatially varying spectral intensities into a representable image. In doing so, the simulator replicates both the brightness and color impression, aiming to create a realistic simulation of the patient's perspective. The following equation models the dispersive behavior of both the eye and the IOL:

$$\begin{array}{c} n\left(\lambda \right)=\;A+\;\frac{B}{\lambda^{2}-\lambda_{0}^{2}} \end{array}$$

In this equation, coefficients *A* and *B* are fitted for each material so the model aligns with the specified center index and Abbe number for the FdC spectral wavelengths. A value of $\lambda_{0}^{2}\;=\;0.014\;µm^{2}$ was chosen for a moderately steep dispersion curve in the violet region, which is a compromise between the Cauchy model ($\lambda_{0}^{2}=\;0.0\;µm^{2}$) and the Herzberger formula ($\lambda_{0}^{2}=\;0.028\;µm^{2}$).^36^ A ray bending approach approximates the diffraction effects inside the eye, as outlined in Appendix C. Convolutional image simulation is also available and explained in detail in Appendix B. The full raytracer is open source and available online.^37^

### Eye Model

The following simulations utilize the Arizona eye model developed by Schwiegerling.^14^ This eye model accurately matches on- and off-axis aberration levels from clinical data and accounts for wavelength and adaptation dependencies. Other researchers have already used this eye model to simulate a virtual phakic or pseudophakic eye.^38^^–^^41^

**Figure 4.**
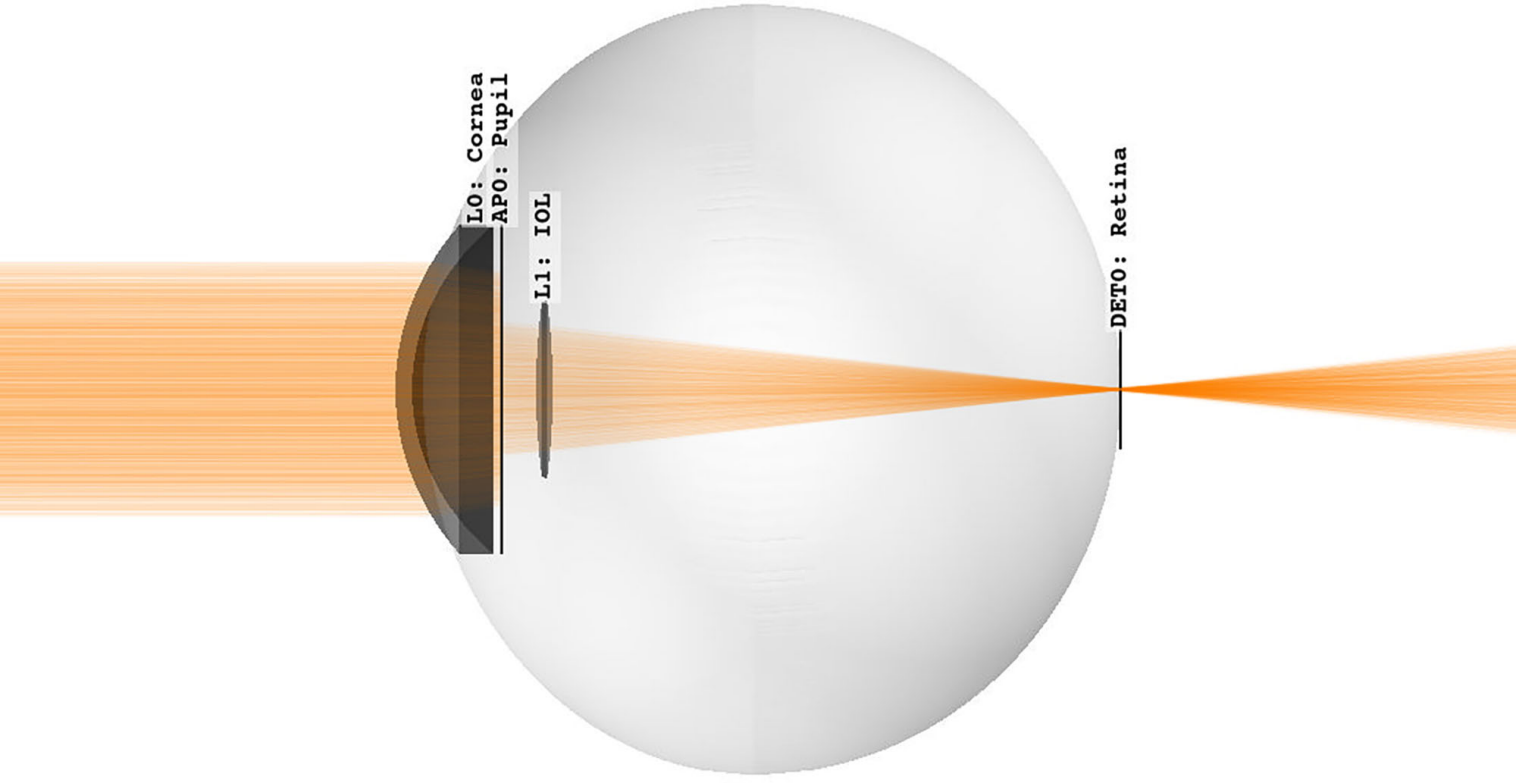
Simulation geometry with the Alcon IQ IOL inside the Arizona eye model.

The IOL is placed at a specific anterior chamber depth (ACD). This value is provided as 3.91 mm for the LuxSmart and 4.23 mm for both Alcon models.^33^ Although the first value worked well for the LuxSmart, the latter values were adapted to 4.10 mm (IQ Vivity) and 4.15 mm (IQ) to better match results of Azor et al.^42^ This small difference can be attributed to differences in the eye model or an imprecise knowledge of the refractive index. Figure 4 illustrates the pseudophakic eye geometry with the Alcon IQ within the raytracer. In our imaging approach, the retina is modeled as a rectangular detector plane. For small angles, such as ±2 degrees, there is only a negligible difference between a plane and a curved retinal surface.

### Color Rendering

For a realistic representation of simulated color images, accurate color rendering is essential. However, the standard red-green-blue (sRGB) color space, widely used in digital media, can only reproduce a limited subset of all human visible colors. To address this limitation, we adopt an approach that preserves image brightness and hue while reducing color saturation. A color transformation with the so-called perceptual rendering intent desaturates the image with a uniform scaling factor and ensures that all colors fit within the target color space. Although this process reduces the overall color intensity, it preserves the relative saturation relationships within the image. Without this adjustment, incorrect saturation ratios could lead to perceptual brightness changes due to the Helmholtz-Kohlrausch effect. The specific computation method is detailed in Appendix A, which also provides an overview of various color spaces.

Printed media have a different, yet also limited color space. For optimal color representation, it is recommended to view this publication on a digital device with high color accuracy and high sRGB coverage.

### Image Simulation

Photic phenomena are effectively represented through the raytracing of pinhole images. Real-life equivalences are the headlights of a car or small dots on a monitor screen. The pinholes are modeled as a circular, Lambertian radiator and emitting a D65 daylight spectrum. The simulations are performed with 60 million rays for each pinhole image. All images were rendered in sRGB with the perceptual rendering intent described in the Appendix A.

Next, resolution charts are an effective method to illustrate the impact of lens model and viewing condition. These types of visualizations are the most accessible for potential patients, as they intuitively demonstrate the effects of acuity and aberrations. The target simulation uses a modified version of a digital recreation^43^ of the US Air Force (USAF)-1951 resolution test chart.^44^ The chart covers a larger field of view and has significantly more structural details than the pinhole. Achieving a similar image quality requires a considerably higher number of rays. Furthermore, even more rays are required to simulate a bright image background. Hence, polychromatic image convolution is utilized to generate the target image, which reduces computational effort and simultaneously minimizes sampling noise. This methodology is described in Appendix B.

A pupil-dependent assessment offers a more precise understanding of both day and night vision scenarios. In the photopic case, the text and lines are rendered black on a white background, whereas in the mesopic case, white text on a black background is simulated. The distinction between the two scenes is crucial, as aberrations affect the image differently in each context. Photic phenomena are much more prominent against a black background, where the aberrations cause an enlargement of structures. Conversely, on a white background, dark structures appear to shrink, and their contrast diminishes significantly.

## Results

### Pinhole Images

**Figure 5.**
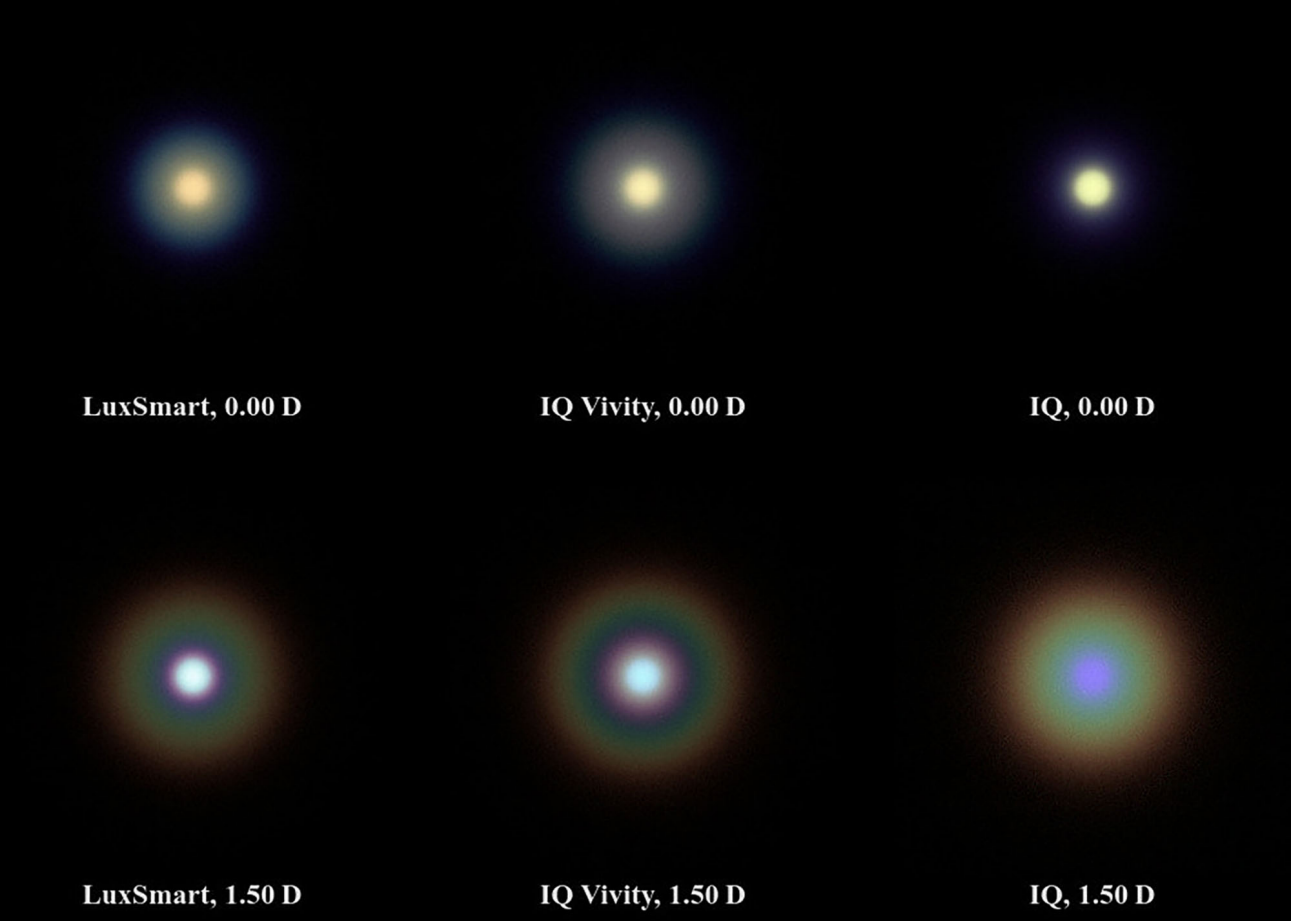
Polychromatic simulation of a 3.4 arcmin pinhole for a 3.0 mm photopic pupil.

**Figure 6.**
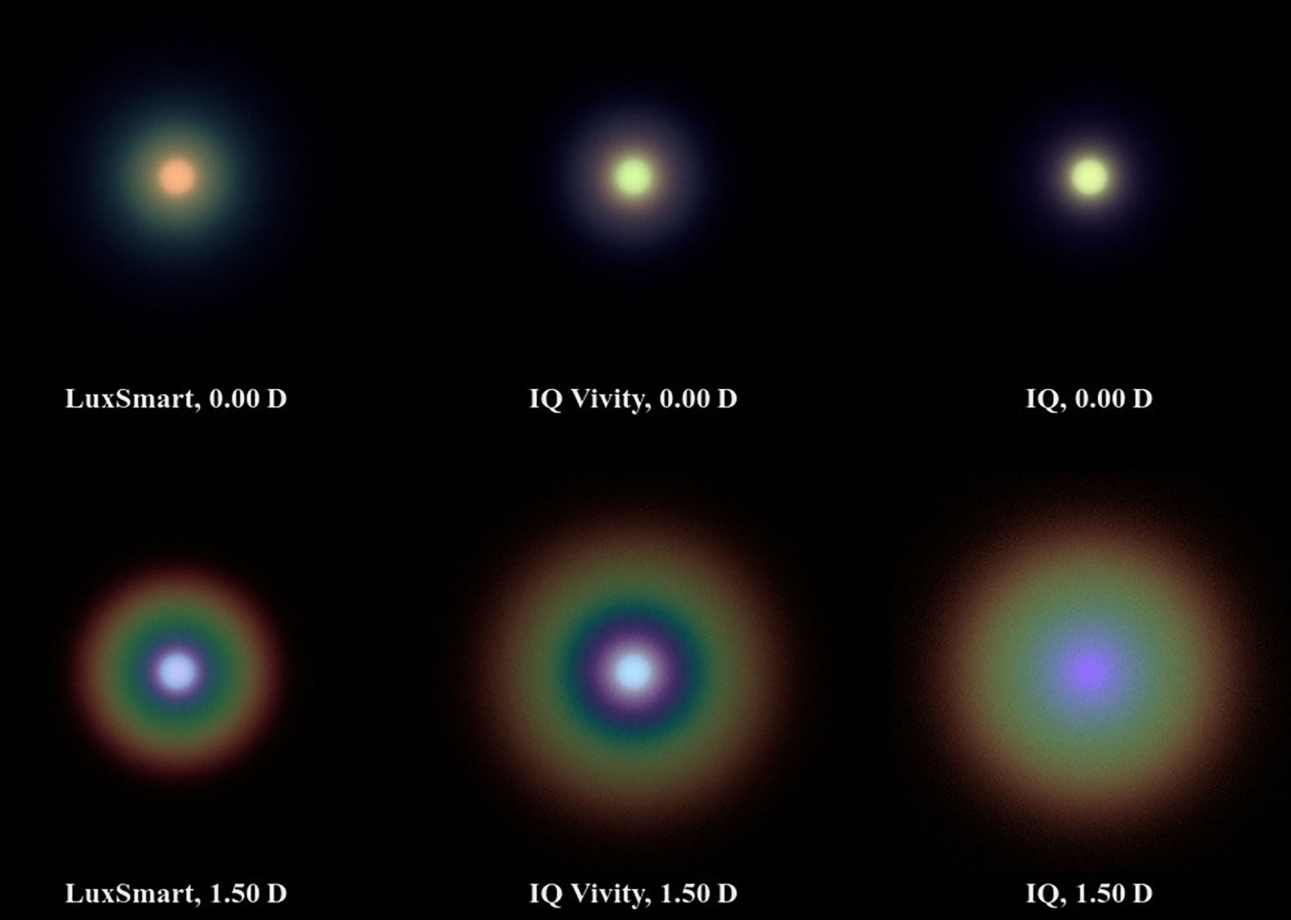
Polychromatic simulation of a 3.4 arcmin pinhole for a 4.5 mm mesopic pupil.

Figure 5 shows the pinhole image results for a pupil diameter of 3.0 mm and Figure 6 for 4.5 mm. Similar to the works of Azor et al.^42^^,^^45^ the pinhole occupies a visual angle of approximately 3.4 arcmin. This corresponds to the size of a 10 cm diameter headlight at 100 m viewing distance (approximately 0.0 D) and a dot of 0.67 mm diameter at 0.67 m (equal to 1.5 D) monitor viewing distance. High resolution versions without potential color artifacts from the proofing process are available in an external repository.^46^

In the photopic situation depicted in Figure 5, the monofocal IQ lens shows the pinhole sharply with no noticeable glare in the distance vision case. However, both LuxSmart and IQ Vivity exhibit significant glare, with the Alcon lens showing slightly more. In intermediate vision, the image produced by the monofocal lens becomes completely out of focus. The IQ Vivity lens presents a halo similar in size to its monofocal counterpart, but contrasts with a slightly defocused pinhole image at its center and a darker ring surrounding it. The LuxSmart lens renders the pinhole more sharply than the IQ Vivity lens, with noticeably less glare. Whereas in the far vision scenario the pinhole appears slightly greenish or orangish for all lenses, in the intermediate scenario it takes on a more bluish hue. At 1.50 D, a distinct color gradient in the halos and glares suggests the presence of chromatic aberration.

In the mesopic simulation in Figure 6, the monofocal lens exhibits slight glaring in distance vision and a more defocused image for the intermediate range compared to the photopic case. The IQ Vivity produces less glare in far vision compared to the photopic simulation, yet it still remains larger than that of the IQ lens. In intermediate vision, the halo is larger compared to the smaller pupil diameter, yet similar in dimensions to the IQ model.

The LuxSmart demonstrates the largest glare among all three lenses for distant vision but exhibits the smallest glare in intermediate vision. Compared to the photopic simulation, there is a noticeable increase in chromatic aberration for all lenses.

### Resolution Charts

**Figure 7.**
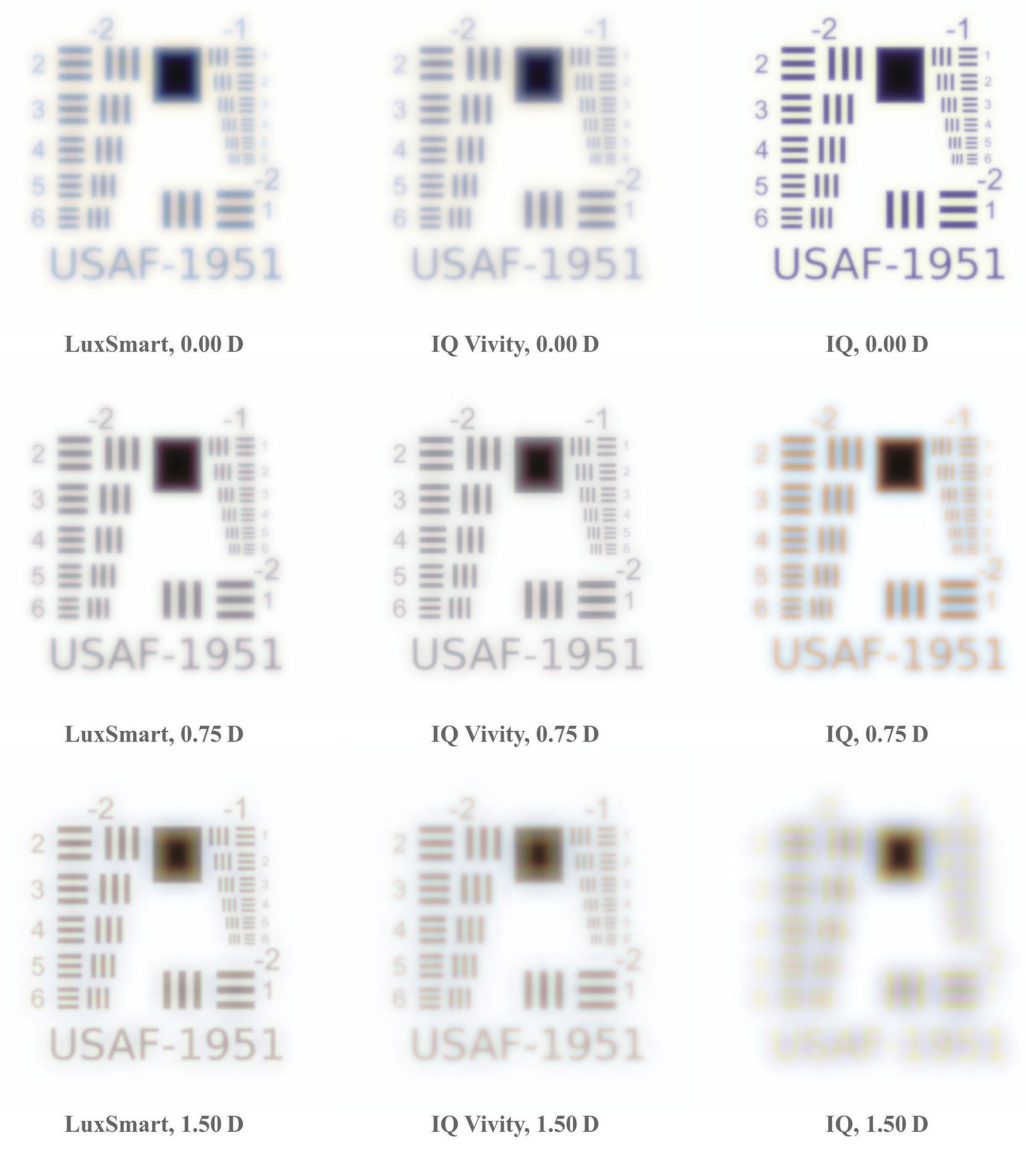
Daylight simulations of the USAF resolution target. The line spacing of element 2 in group −2 corresponds to 10 c/deg.

**Figure 8.**
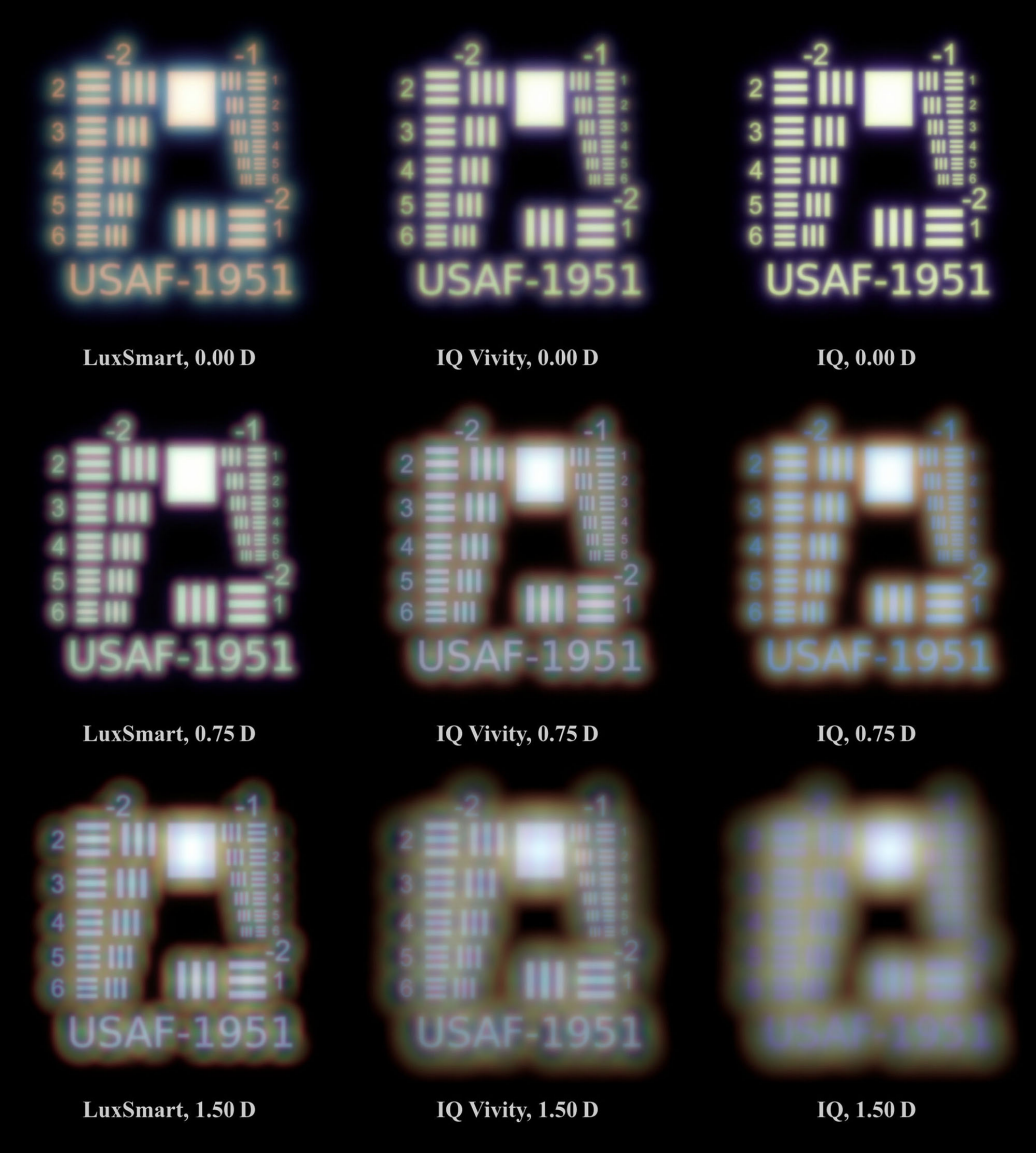
Night-time simulations of the USAF resolution target. The line spacing of element 2 in group −2 corresponds to 10 c/deg.

Resolution charts for a photopic pupil diameter of 3.0 mm are presented in Figure 7, whereas those for a 4.5 mm mesopic pupil can be found in Figure 8. This target occupies a visual open angle of 2 degrees relative to the eye. A point spread function is calculated for object distances of 100 m (≈ 0.0 D), 1.33 m (0.75 D), and 0.67 m (1.5 D). High resolution versions of these figures are available.^46^

Among all three lenses, the monofocal Alcon IQ demonstrates the best performance at 0.0 D in terms of sharpness and minimal aberrations. In the mesopic scenario, a slight amount of glare becomes apparent due to residual spherical aberration. For the other two vergences, the image becomes progressively more defocused, irrespective of pupil diameter. At 0.75 D, only some lines are discernible, and at 1.50 D, no lines are recognizable.

The IQ Vivity lens reproduces nearly all lines for distance vision under photopic conditions, although its performance in terms of acuity and contrast is inferior to that of the monofocal model. At 0.75 D, there is a slight increase in acuity. However, at the highest vergence, sharpness declines and brightness fringes become significantly more pronounced. In the mesopic scenario, the target remains nearly as sharp as with the monofocal model, although a slight glare is seen. However, as vergence increases, glare becomes more pronounced and sharpness significantly diminishes. At 1.50 D, the lines in group −1 are barely distinguishable. At this point, there is a clear overlay of a nearly in-focus blue image and a blurred, greenish image.

The LuxSmart lens produces similarly sharp images across all vergences in photopic vision, with the best performance at 1.50 D, despite the presence of large brightness fringes. In the mesopic case, acuity peaks at 0.75 D. Elements of group −1 remain recognizable across the other two vergences, however, there is an increase in glaring. This is especially true at 1.50 D, where significant chromatic aberration is evident.

## Discussion

### Image Quality

Both EDoF lenses clearly demonstrate an increased depth of focus compared to the monofocal model. As illustrated in Figures 7 and 8, at vergences of 0.75 D and 1.50 D, both lenses exhibit significantly more acuity than the monofocal reference model. Whereas the monofocal lens provides superior acuity at 0.0 D, the EDoF models still deliver sufficient image quality with only a low amount of aberrations. Another important aspect is the presence of glares and halos, which are best observed in the pinhole images in Figures 5 and 6. The EDoF lenses render the pinhole more sharply at shorter distances, and the brightness fringes are of the same or smaller size with reduced intensity. Thus, both the IQ Vivity and LuxSmart not only provide distinctly improved intermediate vision but also display comparable levels of visual disturbances. Clinical studies confirm these findings, indicating both enhanced intermediate vision and similar photic phenomena for these lenses compared with a monofocal intraocular lens.^47^^–^^50^

The Table shows subjective visual quality scores summarizing the resolution chart findings. The two lenses differ in several aspects. The IQ Vivity exhibits an EDoF character under daylight conditions, but behaves more like a monofocal lens under night conditions, as the far vision image is nearly free of aberrations. Nonetheless, a partial EDoF effect remains, because intermediate vision acuity does not decrease as sharply when compared with the monofocal lens. For mesopic vision, the glare is almost identical to that of the monofocal model. This similarity is due to the outer lens areas having an almost identical power profile, as detailed in the previous publication.^32^

**Table.** Authors’ Assessment of Image Sharpness, Contrast, and Photic Phenomena

		LuxSmart	IQ Vivity	IQ
Photopic	0.00 D	+ +	+ +	+ + +
	0.75 D	+ +	+ +	o
	1.50 D	+ +	+	–
Mesopic	0.00 D	+ +	+ + +	+ + +
	0.75 D	+ + +	+ +	o
	1.50 D	+	o	–

Listed as visual quality scores, ranging from – to + + +.

With the LuxSmart during day vision, all three vergences demonstrate high image quality. However, in mesopic conditions, a vergence of 0.75 D stands out with the sharpest vision. At night, the LuxSmart outperforms the IQ Vivity in terms of depth of focus, despite exhibiting more glare in the distance vision image.

These findings align with the positive characteristics that manufacturers attribute to their lenses, as described in the previous article.^32^ Whereas one advantage of the IQ Vivity is its nearly aberration-free performance for night-driving, the LuxSmart excels with an EDoF, irrespective of the lighting conditions.

### Color Effects

Previous images exhibit distinct colored regions, which result from wavelength-dependent and dispersive behavior. A green narrowband stimulus would only reproduce the green-containing areas without other colors such as red and blue. This clearly demonstrates the importance of incorporating the entire human-visible spectral range in image formation.

**Figure 9.**
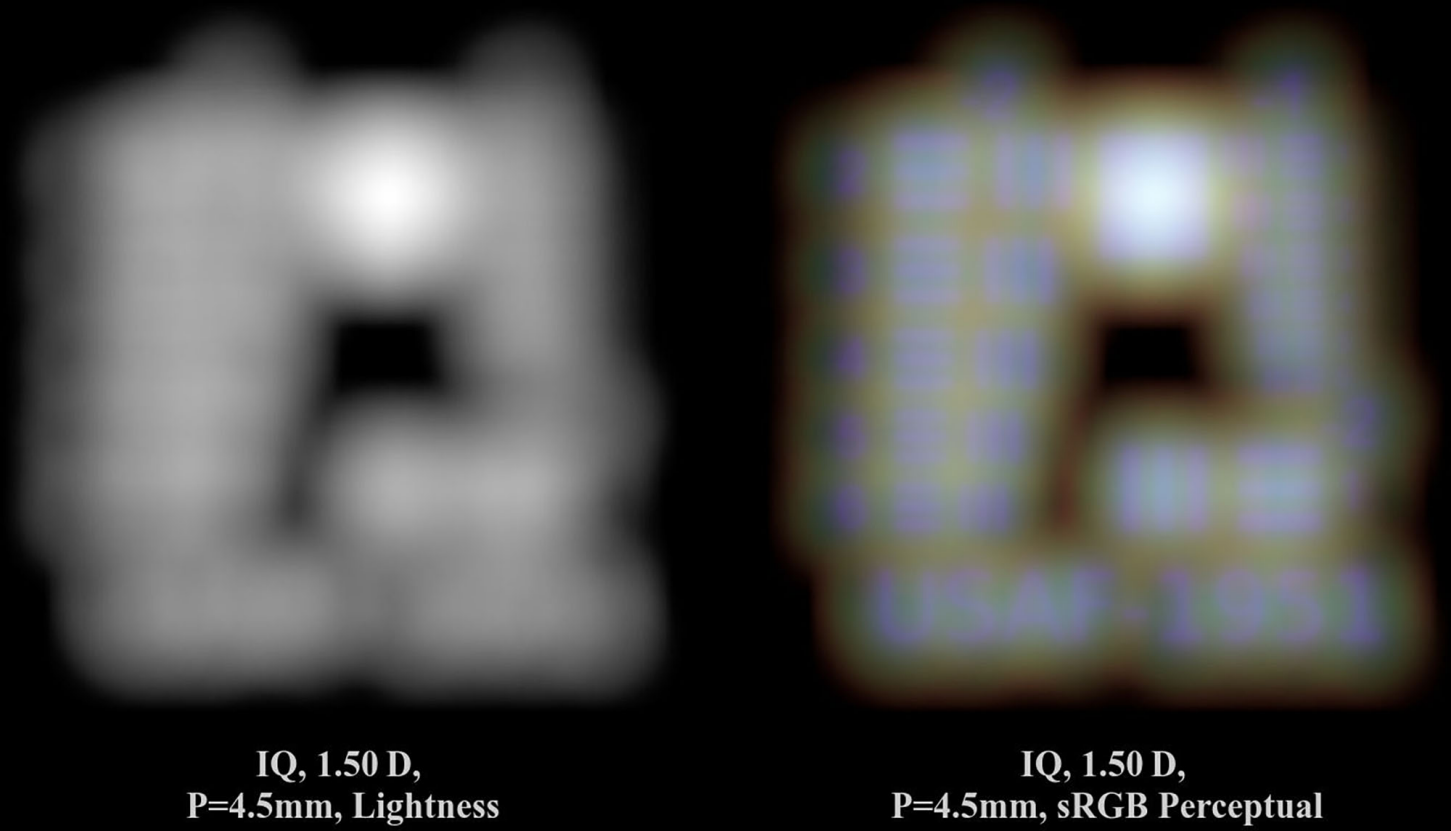
Perceptual difference between a lightness and color (sRGB Perceptual RI) image.

Color significantly contributes to the subjective visual impression, as structures are more easily distinguished through color contrast. This scenario is depicted in Figure 9. The simulation of the Alcon IQ from Figure 8 for pupil diameter *P* = 4*.*5 mm and vergence 1.50 D is presented using only lightness information on the left, and including color information on the right side. Whereas, in the left, the image is only blurry, uniform areas are visible, the right image reveals several distinct elements. The large upper square is apparent, as well as the rough outlines of numbers and group elements. This is possible because the blue light components are nearly in focus, which is also evident in the pinhole image in Figure 6. In reality, the visual impression would be even stronger, as an image with reduced chroma is displayed here.

**Figure 10.**
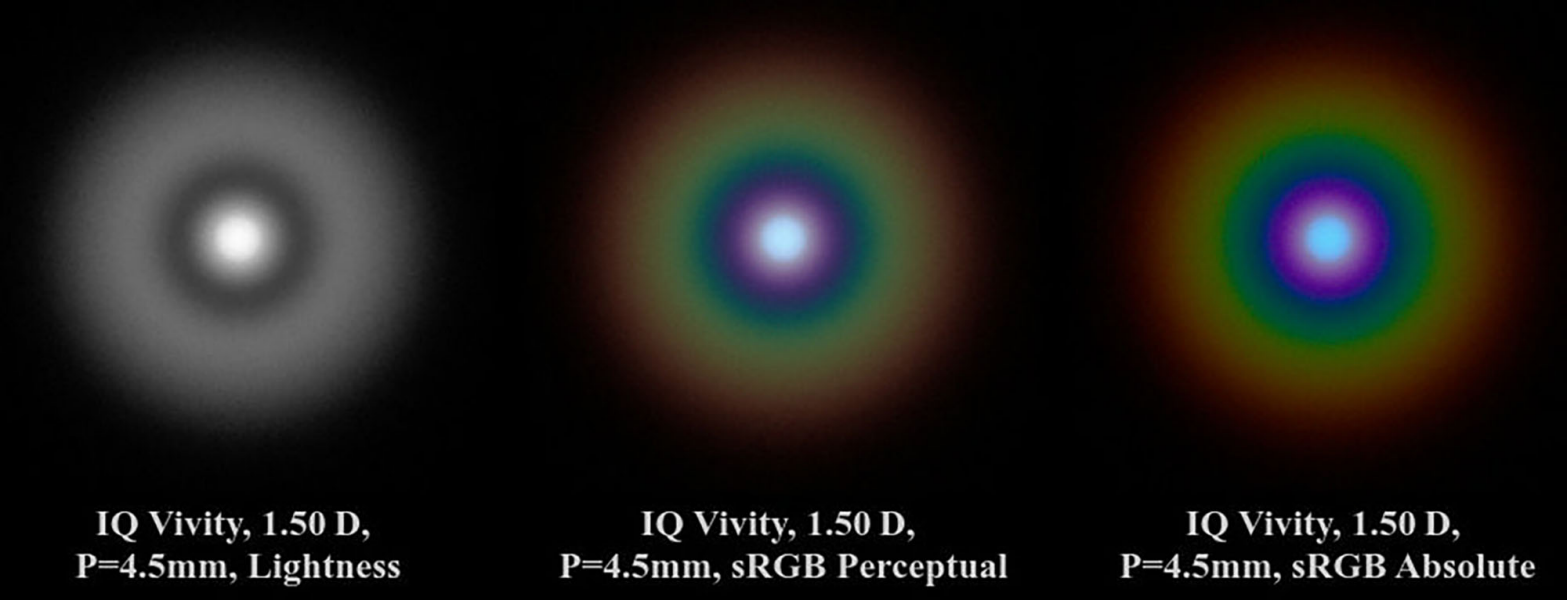
Different color representations for the IQ Vivity pinhole simulation.

As outlined in Appendix A, the type of color conversion and visualization is crucial for results close to reality. Figure 10 shows the important role of rendering intents (RIs). The pinhole image of the Alcon IQ Vivity from Figure 6, for the case of a 4.5 mm pupil and 1.50 D vergence, is displayed in 3 different representations. Going from left to right, these are a lightness image, sRGB with absolute, and perceptual RI. The perceptual RI image correctly reproduces the lightness, whereas the right image deviates noticeably. This is especially visible in the blue ring, which appears much brighter due to its saturation and the Helmholtz-Kohlrausch effect. The sharp edge of this blue ring is due to a high saturation gradient and has no basis in reality. Last, the absolute colorimetric rendering intent also incorrectly reproduces the lightness ratio between the bluish core and the halo.

### Comparison With Earlier Work

#### Pinhole Images

Azor et al.^45^ conducted a direct comparison of pinhole images for the LuxSmart and IQ Vivity lens. These findings are also available in a brochure published by Bausch & Lomb.^51^ The experimental setup, including pupil diameter, viewing angle, and vergences, is consistent with the mesopic simulations depicted in Figure 6, except for a narrowband green light source. Consistent with our findings, the LuxSmart exhibits slightly brighter and larger glare compared to the IQ Vivity lens for distance vision. In addition, under intermediate vision conditions, the Bausch & Lomb lens presents a greater degree of glare compared to the 0.0 D focus, and the IQ Vivity shows a significant halo. In another study by Azor et al.,^42^ their Figure 2 presents the photopic scenario for distance vision. In this figure, both lenses clearly depict the pinhole, yet the glare is significantly more pronounced for the IQ Vivity lens. The results align with the findings of the work at hand.

Fernández-Vega-Cueto et al.^52^ conducted measurements of pinhole images for the IQ Vivity lens. In their paper, it is evident that for far vision, the glare with a 3.0 mm pupil diameter is greater than observed for 4.5 mm, which is consistent with our results.

In the work by Baur et al.,^53^ a comparison of disturbing effects for a 4.5 mm pupil is presented. The IQ Vivity lens displays slightly larger glare than the IQ lens from Alcon. However, in both instances, the disturbing effects are minimal. These observations conform with our findings and align with the measurements provided by Alcon in their “Optical Bench Halo Measurements” brochure section.^54^

The results obtained in the study at hand are consistent with those reported in the previously mentioned publications. Differences in certain aspects can be attributed to the use of a wider spectrum. Furthermore, some works utilize a logarithmic representation of brightness rather than a model based on human visual sensitivity.

#### Resolution Charts

Oltrup et al.^55^ conducted simulations of resolution target images using the measured modulation transfer function (MTF) of the Alcon IQ. Of particular relevance is Figure 7B, which depicts the results for a 4.5 mm pupil and an object angle of 2 degrees. Although the assessments are monochromatic, the simulation conditions are otherwise comparable. The corresponding results in Figure 8 show minimal color effects, therefore allowing for direct comparison of the images. Overall, the simulations convey a similar impression of sharpness.

The study by Schmid et al.^31^ presents a comparison between LuxSmart and IQ Vivity lenses for distance vision, with Figure 4a being particularly relevant to our analysis. Our findings, as presented in Figures 7 and 8, align with theirs by demonstrating similar sharpness for both lenses when the pupil diameter is 3 mm. At a pupil size of 4.5 mm, the IQ Vivity lens clearly outperforms the LuxSmart, which exhibits noticeable blur.

Polychromatic target imaging is explored in the works of Łabuz et al.^24^ and Montagud-Martínez et al.^26^ Although these studies examine different lens models, they reveal the general dispersive properties of IOLs. In some figures, a double image with a white-greenish core and a violet glare is apparent, whereas others display combinations of white-yellow to blue, or white-cyan to red.

The LuxSmart lens product brochure^51^ provides an area of MTF measurement for a 3 mm pupil and different defocus values, comparing it to the IQ Vivity lens. This comparison aligns with the analysis in Figure 7, showing comparable acuity at vergences of 0.0 D and 0.75 D, and demonstrating superior sharpness for the Bausch & Lomb lens at 1.5 D.

The work by Azor et al.^42^ provides an area of MTF values for the two EDoF lenses across a wide defocus range in Figure B. In the figure, the LuxSmart lens achieves a higher value at 1.5 D for a 3.0 mm pupil, while exhibiting values comparable to those of the IQ Vivity lens at 0.0 D and 0.75 D. Under mesopic conditions, the IQ Vivity records a higher area MTF value at 0.0 D but falls short compared to the LuxSmart at the other two vergences. The sharpness and clarity of the line groups mirror the behavior observed in our simulations.

### Limitations

The previous work^32^ discusses the data quality of the lens models. Besides the described deviations in surface shape and refractive index, the results are also influenced by the ACD constant. Although the equivalent constants inside the Arizona eye model are unknown, the selected values most certainly fall inside the expected postoperative positional fluctuation range. All these aforementioned errors lead to changes in global and local optical power, therefore influencing the beam path, shape of the point spread function (PSF), and hence the retinal image.

Differences to the standard setup further complicate the comparison between in vitro measurements and this work's results. Both models differ not only in geometry and materials, but in measurement conditions and procedure as well. Earlier versions of the ISO 11979^29^ demanded a green, narrowband stimulus instead of a broadband spectrum. Furthermore, the object distance is not changed by moving the object, but instead approximated by moving the detector.

The simulator developed does not replicate the complete physics of light. The transmission behavior of the eye and the IOL was not simulated, nor was the influence of stray light or multiple reflections. However, it is to be expected that these influences have only a minor effect on image formation under these experimental conditions. The influence of wave optics is only approximated. Raytracing with the HURB approach from Appendix C implies that the aperture is the primary source of diffraction within the eye and the lens shape does not contribute significantly. The smooth profiles of the monofocal lens and the LuxSmart do not indicate any shape-induced diffraction effects. However, the IQ Vivity features a wavefront shaping design that consists of a more complex annular structure with multiple linear segments.^30^^,^^32^ But upon closer inspection, the minimum width of 100 µm and height differences of 1 µm correspond to a small angular change of 0.6 degrees relative to the base profile. The aperiodic design with wide, shallow segments and smooth transitions similarly suggests that significant diffraction effects are unlikely. Based on these characteristics, a geometric-optical description should suffice for accurate simulation results. Nevertheless, we cannot conclusively exclude a slight impact of wave-optical effects on the through-focus energy distribution. But such deviations are expected to be smoothed out due to additional aberrations, especially spherical aberration and chromatic dispersion.

It is worth emphasizing that a simulation-based approach cannot replace clinical studies. Only in a real eye can the effects of its true geometry be determined, including the exact postoperative position and alignment of the IOL. Last, another missing but indisputable factor for visual outcomes is neuroadaptation.

## Conclusions

This work showcased the method and the results for simulating realistic retinal images. It became evident how relevant the wavelength-dependent behavior is, together with the importance of color in human perception.

The simulation-based approach has proven to be a valuable tool in understanding IOLs. The developed methods successfully characterize IOLs and highlight the differences between two EDoF lenses. This includes technical aspects such as surface structure, refractive power profile, and beam path, as well as the visual impressions through pinhole images or resolution charts. Whereas technical data are crucial for developers or ophthalmologists, simulated images provide the most accessible and comprehensible references for patients.

In the future, the influences of decentration, variation of the anterior chamber depth, or tilt could also be investigated. Another possibility is to simulate a broader range of lenses or tailor the simulation to a patient's specific eye geometry. The simulation offers such flexibility, providing even more application opportunities. Thus, this approach serves as a valuable complement to other methods, offering additional insights into the functionality of IOLs.

## Figures and Tables

**Figure A1.**
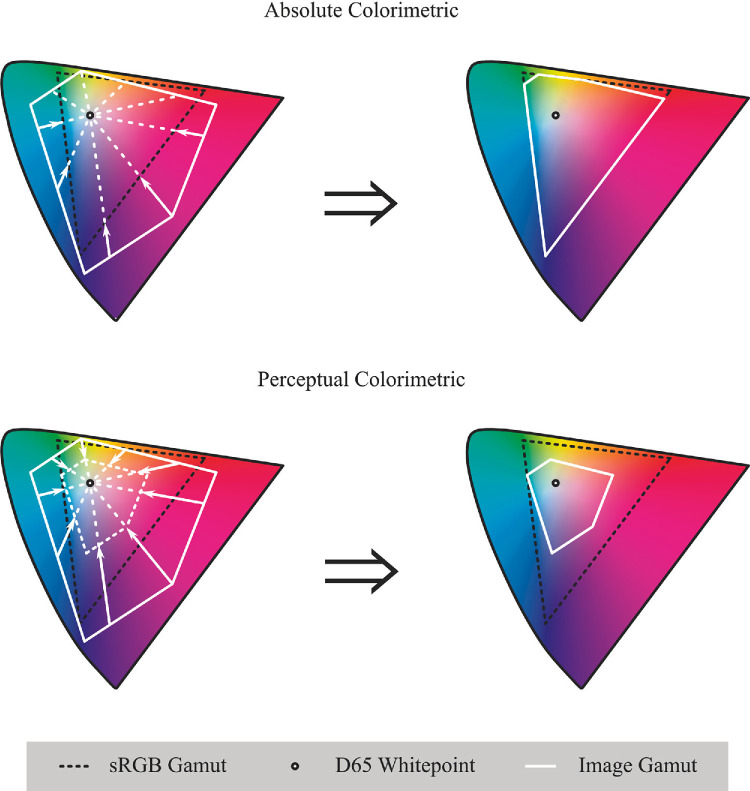
Visualization of absolute and perceptual rendering intent inside the CIELUV UCS diagram.

**Figure A2.**
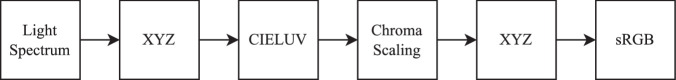
Required steps from light spectrum to sRGB with perceptual rendering intent.

## Appendix A. Appendix A: Details on Color Processing

Accurate color rendering requires careful handling of color space operations. Therefore, it is crucial to thoroughly understand the difference between various color systems.

One of the earliest color spaces developed for describing brightness and color stimuli was the CIE 1931 colorimetric system.^58^ It introduces the CIE 1931 standard colorimetric observers^59^ that define three color matching functions for a narrow field of view of 2 degrees. A light spectrum is weighted according to these functions and integrated over the visible wavelength range to calculate the tristimulus values X, Y, and Z. These constitute the CIE XYZ color system, a device-independent model that encompasses all human-visible colors.

Whereas the system maintains linearity to the light intensity, its components do not create a color space that is linear in terms of human vision. Color appearance models (CAMs) aim to achieve precisely such a perceptually uniform color space. They describe colors through perceptual aspects such as brightness, hue, and saturation, while taking into account chromatic adaptation and different viewing conditions. Widespread CAMs include CIE 1976 CIELUV^60^ and CIELAB,^61^ as well as the modern CAM16-UCS.^62^ Although the latter provides a more accurate description of color vision,^63^^–^^65^ it has a higher mathematical complexity and is less established. For our application, the colorimetric properties of CIELUV and CIELAB are sufficient. In both spaces, the achromatic component, called lightness, closely aligns with the photopic human perception of relative brightness. The primary difference between CIELUV and CIELAB lies in the mapping of color components. In imaging applications, CIELAB is most commonly used because it offers straightforward methods for calculating chromatic adaptation as well as color or brightness differences.^66^ Conversely, CIELUV provides a saturation equation and defines a chromaticity diagram, which represents all possible colors independent of luminance. CIELAB is recommended for the colorant industry, whereas CIELUV is a suitable choice for display industries.^63^

Despite the convenience of CIELAB and CIELUV for color characterization and perceptual calculations, they are not ideal for technical uses of color. In such cases, the standard red-green-blue (sRGB)^67^ color space is used instead. Its primary advantage is the possibility to mix and display colors as a linear combination of three color primaries. Given the reliance of digital media on output devices such as displays, it is unsurprising that sRGB has become the standard color space in this domain. A major disadvantage, however, is that not all colors can be displayed, especially those with strong saturation. Figure A1 illustrates the CIELUV chromaticity diagram, where the colored area includes all visible colors, and the triangle corresponds to the limited sRGB gamut. In color science, the term gamut refers to the range of colors that a particular space can represent. As an sRGB image, Figure A1 is affected by the same limitations of this color space: This explains why highly saturated cyan blue is absent and why saturation appears to remain constant outside the triangular gamut area. To effectively translate colors from one gamut to another, a suitable method must be used. Terms often used in this context include *gamut mapping*, *gamut clipping*, or *gamut compression*. Many software solutions allow for parametrization of this process by setting a rendering intent (RI), which determines which properties should be preserved at the expense of others. A naive, yet widespread approach is to clamp color components to fit into the value range.^68^ But this technique generally alters hue, saturation, and lightness simultaneously.

Here, the focus will lie on two improved rendering intents: *absolute colorimetric* and *perceptual colorimetric*.^69^ In our implementation, these are dimensioned such that the white point is maintained, and only the saturation is adjusted, whereas hue and lightness remain constant. Changing the saturation while keeping the lightness constant is effectively a change of chroma, which describes the chromatic difference to a neutral gray with the same lightness.^70^^,^^71^ Hence, the rendering intents are best described as chroma clipping and chroma scaling. Figure A1 portrays both rendering intents. In the absolute rendering intent, colors are projected toward the white point onto the edge of the gamut, whereas colors within the target gamut remain unchanged. However, this approach does not preserve chroma ratios, leading to slight perceptible changes in brightness due to the Helmholtz-Kohlrausch effect. In contrast, the perceptual rendering intent downscales the saturation uniformly so that all colors fit within the target gamut. To achieve accurate results, chroma scaling must be performed in a perceptually uniform representation, such as the CIELUV 1976 uniform chromaticity scale (UCS) diagram. The drawback of this intent is the desaturation of the image, as no absolute chroma values are preserved.

In this work, the perceptual rendering intent is applied to the image because preserving brightness relationships is a priority. To ensure better comparability across multiple images, a uniform scaling factor was selected that is effective for all images. The processing steps from light spectrum to sRGB are shown in Figure A2. Conversions to XYZ are required, as the conversions for other color spaces are typically defined from this space.

Although most technical devices strive to cover at least the entire sRGB space, they often fall short due to technical limitations. Additionally, a further color space conversion occurs for printed media, which is determined by the cartridge colors. This conversion can further distort results and decrease the range of available colors.

## Appendix B: Polychromatic Image Convolution

For small angles, an optical setup can be approximated as a linear system. In such a system, imaging is simulated by convolving the object image with the system's point spread function (PSF). However, because the PSF depends on the lateral position on the object plane, it is only applicable within a small angular region. This condition is satisfied, because we only use paraxial rays within a ±2 degrees angular range.

Compared to conventional raytracing, convolution significantly reduces processing time because the effect of the PSF is applied in parallel to all object positions. Additionally, the resulting image contains minimal sampling noise. Monte-Carlo methods are prone to this noise because only a random subset of all possible ray properties is simulated. Although the PSF image does include some sampling noise, most of it exists on a smaller spatial scale compared with the object's structural components. Moreover, the convolution operation acts as a blurring filter, which reduces noise to a large extent.

To incorporate color information, both the object and PSF must be convolved separately for each wavelength. Simply convolving the color space coordinates is insufficient because many spectral distributions can result in the same color appearance, due to a phenomenon known as metamerism. Consequently, different spectra can produce distinct PSFs, meaning that the relationship between color and PSF is not unequivocal without some additional constraints. The process of creating multicolored images through convolution has been explored by Ravikumar et al.,^72^ building upon the findings of Barnden.^73^ Ravikumar et al. demonstrate that convolution using a polychromatic PSF, which is the sum of multiple per-wavelength PSFs, yields correct results only if the object is spectrally homogeneous. Spectrally homogeneous objects emit the same spectrum everywhere, with variations occurring only in local intensity. Although most natural scenes are spectrally inhomogeneous, man-made objects such as printed or electronically displayed grayscale media meet this criterion.

Because convolution is a linear operation, it must be performed in a color space that is linear with respect to spectral intensities. Therefore, the linear sRGB space is applied. Unlike standard sRGB, which applies a nonlinear gamma correction, linear sRGB is not aligned with human perception but the light's intensity. To ensure color processing for all colors, negative values are also considered in the convolution operation. These values represent colors outside the sRGB gamut but are still valid. After performing channel-wise convolution, the image is converted back into normal sRGB, while applying gamut mapping using the perceptual rendering intent.

To simulate the image formation of a spectrally homogeneous black-and-white object, it is necessary to select a spectrum that corresponds to the white point in the color space. The white point of the sRGB space is defined by the D65 standard illuminant,^74^ which represents average daylight with a color temperature of 6504K. Due to its widespread use and relevance in everyday scenarios, we have chosen D65 for our simulations. The PSF is generated by tracing this specific spectral distribution from a point source to the detector plane. The object itself is modeled as spectrally homogeneous, characterized by varying grayscale values that represent the intensity of the emitted spectrum. To create the simulated image, the distribution is then convolved with the PSF on a per-channel basis.

This PSF is valid only for the same object and image distance it was generated for. It must be convolved using the correct dimensions for both the image and PSF, along with the system's magnification factor.

## Appendix C. Appendix C: Modeling Edge Diffraction

Raytracing, as a purely geometrical-optical approach, does not account for diffraction and interference effects. This limitation can lead to inaccurate predictions of image quality, particularly around the focal region and in diffraction-limited systems. To overcome this, a procedure called Heisenberg Uncertainty Ray Bending (HURB) was implemented to simulate edge diffraction effects. The concept originates from the work of Carlin^75^ and Heinisch and Chou^76^ and was later expanded upon by Likeness^77^ and Coffey et al.^78^ The core idea involves applying a directional variation to the ray's path within the aperture plane. This variation is determined by the ray's distance to the aperture edges and the principle of position-momentum uncertainty from quantum mechanics. The result is a spatially varying angular spectrum that propagates through the rest of the optical system.

Several authors demonstrated HURB's ability to accurately model the blurring tendency in both near- and far-field scenarios.^76^^–^^80^ In this work, HURB is used to correctly model the resolution limit of the eye and the beam profile around the focus region. Similarly, Lian et al.^81^ previously modeled the eye's diffraction limit by applying HURB at the pupil. Implementation details and comparisons between simulation and theory are available in the simulator's documentation.^82^

There are extensions of HURB that incorporate the light's phase to also simulate interference effects.^79^^,^^80^^,^^83^ However, these were not applied in this work. Admittedly, this results in only modeling the asymptotic blurring behavior of diffraction, but not the gaps and regions of destructive interference. However, due to the eye's incoherent imaging, the EDoF lenses’ elongated focal region, and the presence of chromatic aberration, we expect the gaps to be overlapping and less visible.

Last, HURB approximates edge diffraction effects only and cannot simulate diffractive structures such as the kinoform profile of a typical multifocal lens. As a consequence, the modeling of non-refractive IOLs requires other computational methods.
